# Obsessive-compulsive disorder presenting with musical obsessions in otosclerosis: a case report

**DOI:** 10.1186/1752-1947-8-384

**Published:** 2014-11-24

**Authors:** Lucrezia Islam, Silvio Scarone, Orsola Gambini

**Affiliations:** 1 Psychiatry, University of Milan Medical School, Ospedale San Paolo, Psichiatria Ovest, Via A Di Rudini 8, 20142 Milan, Italy

**Keywords:** Hearing loss, Music, Obsessions, Otosclerosis

## Abstract

**Introduction:**

Musical obsessions consist of intrusive recollections of music fragments that are experienced as unwanted. Otosclerosis is caused by an abnormal bone homeostasis of the otic capsule and represents a frequent cause of hearing impairment. Many conditions causing hearing loss have been associated with musical hallucinations, but the association between musical obsessions and hearing loss is frequently overlooked.

**Case presentation:**

We present the case of a 51-year-old Caucasian woman with a history of obsessive-compulsive disorder who developed musical obsessions soon after being diagnosed with otosclerosis. She was referred to our obsessive-compulsive disorder outpatient unit by her general psychiatrist. At the time of our first evaluation, she had severe musical obsessions that interfered with her social functioning and made her unable to follow conversations. She was started on 40mg of paroxetine and 2.5mg of aripiprazole, which led to significant improvement of her symptoms and of her social and work functioning.

**Conclusions:**

To the best of our knowledge, this is the first report of musical obsessions in a patient with hearing loss due to otosclerosis and a history of obsessive-compulsive disorder. This case suggests that a differential diagnosis of obsessive-compulsive disorder should be carefully considered in patients with hearing impairment who complain of involuntary musical imagery, especially in those patients who have a previous history of obsessive-compulsive disorder.

## Introduction

Obsessive-compulsive disorder (OCD) is defined by the presence of obsessions, compulsions, or both. An *obsession* is defined as an unwanted intrusive thought, doubt, image, or urge that repeatedly enters the mind and causes marked distress and anxiety. Obsessions are distressing and ego-dystonic; that is, they are inconsistent with the person’s self-image [1]. The person usually regards the intrusions as unreasonable or excessive and tries to resist them. Musical obsessions are one of the many clinical features of OCD.

Many people may experience involuntary musical imagery (INMI) or "earworms". These terms describe the spontaneous recall and replay of musical imagery within the mind’s ear that repeat in an involuntary loop [2]. Musical obsessions consist of intrusive recollections of music fragments (that is, music running through one’s mind), which the patient experiences as unwanted and tries to suppress [3-7]. There have been no epidemiologic studies assessing musical obsessions. According to a recent comprehensive review of 96 papers published on this topic, clinically relevant phenomena involving INMI may be underestimated [4]. The review’s authors proposed that the reasons may be that most previously published reports are of isolated cases, which implies that musical obsessions are a rare condition, and that current assessment methods do not sufficiently probe for such phenomena.

Many conditions determining hearing loss have been associated with musical hallucinations [8,9], which are characterized by perception of musical sounds in the absence of any external source of music [8,10,11]. Otosclerosis, a condition caused by an abnormal bone homeostasis of the otic capsule that frequently results in hearing impairment in white adults [12], has been shown to cause musical hallucinations. The hallucinatory phenomena may arise as a direct consequence of subacute hearing loss caused by otosclerosis, which triggers the auditory cortex [13]. Although the association between hearing loss and musical hallucinations is well known in clinical work, the relationship between hearing impairment and obsessions with musical content, defined as INMI that meets criteria for OCD, may be overlooked. To the best of our knowledge, there are no previous case reports in the literature describing musical obsessions in association with hearing loss. In this report, we describe the case of a patient with otosclerosis and musical obsessions.

## Case presentation

A 51-year-old Caucasian woman was referred to our outpatient OCD unit because of recurrent intrusive musical obsessions. Her previous medical history was unremarkable. She had worked as a secretary and was unemployed at the time of our evaluation. She had no family history of psychiatric, neurological, or hearing disorders.

The patient had had OCD since age 15 years. At the time of onset, her main symptoms consisted of aggressive obsessions (fear that she might harm someone else with a knife) associated with avoidance, mental compulsions (repetition of phrases), and checking. Starting at 30 years of age, she had been treated with several selective serotonin reuptake inhibitors (SSRIs), including sertraline, paroxetine, and clomipramine, with significant improvement of her OCD symptoms. She subsequently relapsed after the medications were withdrawn, but she decided to stay off medication because her symptoms were not as disturbing anymore. In addition to pharmacological treatment, she had undergone cognitive-behavioral therapy (CBT) for more than 1.5 years, but she discontinued it because she thought did not benefit from the treatment.

In 2008, when the patient was 45 years of age, she had started to notice hearing loss, for which her general practitioner referred her to an ear, nose, and throat specialist. He ordered audiometric testing and head computed tomography, and he diagnosed her with otosclerosis on the basis of these test results.

Approximately three months later, the patient had noticed intrusive music playing in her head continuously, making her unable to concentrate on ongoing conversations, books, or any other activity. She described the intrusive music as "terribly annoying", and she said she sometimes tried to suppress the obsessions by voluntarily singing a different tune out loud or in her head or by playing very loud music on her stereo. The intrusive music was sometimes triggered by tunes playing on the radio or on television, similarly to what is described for earworms [14], and she indicated that the music was coming from "inside the head", as opposed to its being perceived from an external source. She stated that the obsessions worsened progressively as her hearing got worse, and she said that they were more frequent and distressing when she was in quiet places, whereas loud sounds or crowded, noisy places seemed to make them a little better. She had trouble falling asleep because of the obsessions, but she had no trouble staying asleep or with early awakening.

She had initially sought help at a general psychiatry outpatient facility. Suspecting musical hallucinations, her psychiatrist prescribed risperidone up to 3mg, which did not lead to any significant improvement of symptoms. Her psychiatrist then correctly diagnosed her with OCD and referred her to our OCD unit.

At her baseline evaluation, her mood was slightly depressed because of the continuous musical obsessions, but she showed no other sign of depression and she had mild anxiety. No other psychiatric symptoms were noted upon examination.

The patient was screened with the Yale-Brown Obsessive-Compulsive Scale (Y-BOCS), the Obsessive-Compulsive Spectrum Self-Report (OBS-SR; lifetime and past month versions), the Brown Assessment of Beliefs Scale (BABS), the Hamilton Depression rating Scale (HAM-D), and the Hamilton Anxiety Rating Scale (HAM-A). Her Y-BOCS score was 36 (obsessions: 20, compulsions: 16), her OBS-SR lifetime score was 80, her OBS-SR past month score was 40, her BABS score was 2, her HAM-D score was 16, and her HAM-A score was 14. The scores indicated severe OCD with good insight.

The patient also underwent psychiatric assessment by means of the Structured Clinical Interview for DSM Disorders (SCID) I and II for major psychiatric disorders and personality disorders. The SCID I revealed a diagnosis of lifetime and current OCD, whereas the SCID II did not reveal any personality disorders. Her personality traits were also assessed by means of the Neo Personality Inventory–Revised (NEO-PI-R). On that test, the patient scored high on neuroticism, particularly regarding the anxiety, self-consciousness, and vulnerability facets. She also scored high on the consciousness domain, particularly on the order and self-discipline domain. Her remaining scores were normal, with the exception of a low score on the openness to new practical experience facet (openness to experience domain).

After the initial evaluation, the patient was started on 20mg of paroxetine, which was then increased to 40mg. We advised the patient to start CBT, but she refused because of her past experience. Six weeks after she was started on paroxetine, her symptoms had improved and her Y-BOCS score had decreased from 36 to 26. After 12 weeks, there was no ulterior improvement and she was still very bothered by the obsessions, so 2.5mg of aripiprazole was added to her regimen as an augmentation agent. Two weeks later, her Y-BOCS score had significantly decreased, going from 26 to 16; her sleep pattern and mood were better; and she reported feeling better. At her three-month follow-up visit, her Y-BOCS score was 15, indicating mild OCD; her HAM-D score was 6 and her HAM-A score was 4.

## Discussion

Musical hallucinations, which are characterized by perception of musical sounds in the absence of any external source of music [8,10,11], have been described in patients with hearing loss [8,9,15] and are a phenomenon similar to visual hallucinations in patients with loss of vision or acquired blindness [8,16]. It is thought that the absence of external acoustic stimuli may stimulate the auditory cortex, which produces hallucinatory phenomena [17,18]. INMI, which consists of earworms and musical obsessions, may also be triggered by the absence of external acoustic stimuli [19]. Earworms are perceptions of spontaneous, repetitive musical sounds in the absence of an external source [2,18], whereas musical obsessions are episodes of INMI that meet criteria for OCD symptoms, meaning that they are recurrent, persistent, intrusive, and time-consuming and cause distress or functional impairment [4].

Although our patient had a positive history of OCD, she had never presented with musical obsessions before the onset of her otosclerosis, and her obsessions appeared to be dramatically worsened by the absence of external stimuli loud enough for her to hear. Remarkably, our patient scored high on neuroticism on the NEO-PI-R. It has been reported that patients with neurotic tendencies may be more prone to earworms [20,21]. We hypothesize that musical obsessions may share common mechanisms with musical hallucinations in patients with hearing impairment and that the reduction or absence of auditory stimuli may stimulate the auditory cortex, triggering these types of obsessions.

In treating our patient, we followed guidelines for the treatment of OCD. Because our patient had only a partial response to SSRIs, which are considered a first-line treatment for OCD, a low-dose atypical antipsychotic had to be added to her medication to achieve clinical remission of her symptoms. CBT is also considered a first-line treatment for OCD and has been reported to have been used successfully to treat musical obsessions [18]. Unfortunately, our patient repeatedly refused CBT. Our patient ultimately reported improvement of her obsessions when listening to different tunes, which acted as competing stimuli. Distraction is successfully used for earworms [22,23], and it has been proposed that it may also be helpful for musical obsessions [4].

## Conclusions

To the best of our knowledge, this is the first report of a patient with musical obsessions presenting with hearing loss due to otosclerosis and with a history of OCD. Our report suggests that a differential diagnosis of OCD should be carefully considered in patients with hearing impairment who complain of INMI, especially in those patients who have a previous history of OCD.

## Consent

Written informed consent was obtained from the patient for publication of this case report and any accompanying images. A copy of the written consent is available for review by the Editor-in-Chief of this journal.

## Abbreviations

BABS: Brown Assessment of Beliefs Scale; HAM-A: Hamilton Anxiety Rating Scale; HAM-D: Hamilton Depression Rating Scale; INMI: Involuntary musical imagery; NEO-PI-R: Neo Personality Inventory–Revised; OBS-SR: Obsessive-Compulsive Spectrum Self-Report; OCD: Obsessive-compulsive disorder; SCID: Structured Clinical Interview for DSM Disorders; SSRI: Selective serotonin reuptake inhibitor; Y-BOCS: Yale-Brown Obsessive-Compulsive Scale.

## Competing interests

The authors declare that they have no competing interests.

## Authors’ contributions

LI examined the patients, conducted the psychiatric assessments and wrote the manuscript. SS provided supervision. OG substantially contributed to the manuscript writing and revision. All authors read and approved the final manuscript.
